# Age, neutrophil lymphocyte ratio, and radiographic assessment of the quantity of lung edema (RALE) score to predict in-hospital mortality in COVID-19 patients: a retrospective study

**DOI:** 10.12688/f1000research.26723.2

**Published:** 2021-01-06

**Authors:** Anggraini Dwi Sensusiati, Muhammad Amin, Nasronudin Nasronudin, Alfian Nur Rosyid, Nanda Aulya Ramadhan, Rofida Lathifah, Eva Puspitasari, Ria Indah Wahyuningtyas, Erika Soebakti

**Affiliations:** 1 Department of Radiology, Universitas Airlangga Hospital, Surabaya, East Java, 60115, Indonesia; 2 Department of Research, Planning and Development, Universitas Airlangga Hospital, Surabaya, East Java, 60115, Indonesia; 3 Medical Faculty, Universitas Airlangga, Surabaya, East Java, 60115, Indonesia; 4 Department of Pulmonology, Universitas Airlangga Hospital, Surabaya, East Java, 60115, Indonesia; 5 Department of Internal Medicine, Universitas Airlangga Hospital, Surabaya, East Java, 60115, Indonesia; 6 Department of School of Health Science, City, University of London, London, EC1V 0HB, UK

**Keywords:** COVID-19 mortality, NLR, RALE Score

## Abstract

**Background: **Available data suggest that case fatality rate of COVID-19 patients in Surabaya is higher than global cases. Thus, it is important to identify risk factors to prevent the mortality. This study aimed to assess the factors associated with hospital mortality of COVID-19 patients, and develop a prediction score based on these findings.

**Methods: **We analyzed 111 patients, who were diagnosed with COVID-19 based on reverse-transcriptase polymerase chain reaction. The following patient characteristics were obtained from records: age, gender, type of symptoms, onset of symptoms, neutrophil lymphocyte ratio (NLR), absolute lymphocyte count, chest x-ray abnormalities, lung involvement, type of lesion, radiographic assessment of the quantity of lung edema (RALE) score, and mortality. Data were analyzed using SPSS 25.0.

**Results **Multivariate analysis showed that age >50 years ( *p*=0.043), NLR score >5.8 ( *p*=0.016) and RALE score >2 ( *p*=0.002) can predict the mortality of COVID-19 patients in the hospital. ROC curve analysis of the score ability to predict mortality showed an area under the curve of 0.794. The cut-off point is 4.5, with a sensitivity of 96.7% and specificity of 49.4% to predict the mortality of COVID-19 patient in the hospital.

**Conclusions **Age, NLR score and RALE score were associated with mortality of COVID-19 patients in the hospital and might be used as a predictor for mortality of COVID-19 patients in health care centre where radiologists are available. The prediction score may be useful for frontline physicians to effectively manage patients with a higher score to prevent mortality.

## Introduction

Cases of pneumonia of unknown etiology were first reported in the city of Wuhan, China, at the end of December 2019. After identification, the etiology of these cases was a new type of coronavirus ( Wang *et al*., 2020). The World Health Organization officially called this coronavirus disease 2019 (COVID-19) ( WHO, 2020). COVID-19 is caused by severe acute respiratory syndrome coronavirus-2 (SARS-Co-V2) virus ( Gorbalenya *et al*., 2020).

Since December 2019, more than 20 million people have been diagnosed with COVID-19, and more than 700,000 have died ( Worldometers, 2020). In Indonesia, 128,776 people have been diagnosed with COVID-19, and 83,710 have died since March 2020 ( Gugus Tugas Percepatan Penanganan COVID-19, 2020). The global case fatality rate (CFR) of COVID-19 in August was 3.7%. In Indonesia, the CFR is slightly higher (4.5%). Meanwhile, the CFR in Surabaya (the second biggest city in Indonesia) is almost twice that of Indonesia for the same period (8.9%). Several factors may associate with the higher CFR such as low testing, low contact tracing and poor COVID-19 register ( Sorci *et al*., 2020). However, finding characteristics and risk factors to predict mortality is of utmost importance in order to prevent the mortality. It is also important to develop a prediction score to assess patients during early stages, when patients receive treatment at tertiary hospitals.

Several studies have found risk factors related to COVID-19 mortality ( Galloway *et al*., 2020; Gupta *et al*., 2020; Jalili *et al*., 2020; Lippi & Plebani, 2020; Zhao *et al*., 2020). However, currently, there is no study that analyzes risk factors for COVID-19 mortality in hospitals for the Indonesian population. Existing prediction scores also based on complex laboratory findings, which are less feasible to be used in low resource health centers. Therefore, the purpose of this study was to assess the demographical characteristic, laboratory and radiological findings associated with COVID-19 mortality in hospital patients and develop a prediction score based on these findings.

## Methods

### Study design and setting

This was a retrospective study that was conducted in the Emergency Department of Airlangga University Teaching Hospital. We included all the patients who fulfilled the inclusion and exclusion criteria, who were admitted for COVID-19 from March 13, 2020 to May 15, 2020. A total of 111 patients met the criteria and included in this study.

We collected data regarding clinical symptoms and the date of onset of symptoms from emergency department medical records.

### Patients

The inclusion criteria for this retrospective study were: (1) patients with a chief complaint of one COVID-19-related symptom, including: fever, dry cough, tiredness, aches and pains, nasal congestion, headache, conjunctivitis, sore throat, diarrhea, loss of taste or smell, a rash on skin, or discoloration of fingers or toes ( WHO, 2020); (2) confirmed SARS-CoV-2 infection by reverse-transcriptase polymerase chain reaction (RT PCR) using nasopharyngeal and oropharyngeal specimens; (3) patients who underwent chest x-ray (CXR) on the day of admission to the hospital.

The following patients were excluded: (1) asymptomatic patients; (2) those with negative results for SARS-CoV-2 infection by RT-PCR.

All patients underwent CXR and laboratory examination on the day of admission to the emergency department.

### Subgrouping of patients

Patients were divided into two groups based on the outcome of the patient: group I (discharged with negative results for SARS-CoV-2 infection by RT-PCR test); and group II (died).

### Data collection

The following patient characteristics were obtained from the medical records: age, gender, type of symptoms, onset of symptoms, neutrophil lymphocyte ratio (NLR), absolute lymphocyte count (ALC), CRX abnormalities, lung involvement, type of lesion, radiographic assessment of the quantity of lung edema (RALE) score, and mortality.

***Image acquisition and evaluation.*** Radiology data was collected from the radiology department. All the patients underwent an anteroposterior projection chest radiography at full inspiration where possible. The results were reviewed by two radiologists (A.D.S, a radiologist with 25 years of experience and E.S, a radiologist with four years of experience) based on consensus.

CXRs were evaluated for the presence of pulmonary alterations, type of pulmonary alterations, and their distribution. CXR alterations that were found specifically in COVID-19 patients were defined according to the Fleischner Society’s nomenclature, available in the Glossary of Terms for Thoracic Imaging ( Hansell *et al*., 2008), as follows:

-     Reticular alteration: a reticular pattern is a collection of innumerable small linear opacities that, by summation, produce an appearance resembling a net

-     Consolidation: as a homogeneous increase in pulmonary parenchymal attenuation that obscures the margins of vessels and airway walls

-     Ground-glass opacity (GGO): an area of hazy increased lung opacity, usually extensive, within which margins of pulmonary vessels may be indistinct

The distribution of pulmonary alterations was classified as lung involvement unilateral (right/left) or bilateral. Other features, such as pleural effusion, were also recorded.

***Radiograph scoring.*** A severity score was calculated to quantify the extent of the infection by adapting and simplifying the RALE score proposed by Warren **et al*.* (2018). A score of 0-4 was assigned to each lung depending on the extent of involvement by consolidation or GGO (0 = no involvement; 1 = <25%; 2 = 25-50%; 3 = 50-75%; 4 = >75% involvement). The scores for each lung were summed to produce the final RALE score.

### Statistical analysis

Statistical analysis was performed using IBM SPSS Statistics Version 25.0. Continuous variables were expressed as mean ± standard deviation values. The frequency of symptoms, laboratory findings and CXR findings was shown as the number of incidence and percentage per cluster of groups. The correlation between the patient characteristic, symptoms, laboratory findings and CXR findings with the outcome was analyzed by logistic regression. We also conducted the Hosmer-Lemeshow test to evaluate the goodness of fit of the scoring model and conducted receiver operating characteristic (ROC) analysis to evaluate the sensitivity and specificity of the model.

### Ethical considerations

This study received ethical approval from the Ethical Committee of Airlangga University Teaching Hospital (approval number 147/KEP/2020). Consent from the participants was waived by the committee.

## Results

### Patient characteristics

A total of 111 COVID-19 patients were evaluated. In total, 72.9% (n=81) of patients were discharged from the hospital, while 27.1% (30) patients died during their hospitalization. There were 45 men (48.6%) and 47 women (51.4%). Average patient age was 51±14.2 years old. The mean age for the patients who died was higher (55±12.8 years) compared to the patients who were discharged (48±14.8 years). Fever (32.6%) and shortness of breath (26%) were the most frequent symptoms for all patients. However, among the patients who died, shortness of breath was the most frequent symptom (40.7%). The mean NLLR score is higher on the patient who died (10.1 ±10.5) compared to patients who were discharged (5.3 ±5.4). In contrast, the mean ALC score was lower in patients who died (1130±252) compared to patients who were discharged (1349±702). Most of the patients showed GGO on their CXR (67.6%). Mean RALE score was higher in patients who died (5.3±2.5) compared to patients who were discharged (2.7±2.7) ( Table 1).

**Table 1.** Patient characteristics.

Variables	All patients (n=111)		Discharged (n=81)		Died (n=30)	
	n	%	n	%	n	%
**Mean age ±SD**	50 ±14.6		48 ±14.8		55 ±12.8	
**Gender**						
Men	54	48.6	39	48.1	15	50.0
Women	57	51.4	42	51.9	15	50.0
**Clinical symptoms**						
Fever	36	32.4	30	37.0	6	20.0
Cough	22	19.8	20	24.7	2	6.7
Fatigue	11	9.9	8	9.9	3	10.0
Shortness of breath	29	26.1	16	19.8	13	43.3
Headache	3	2.7	0	0.0	3	10.0
Abdominal pain	3	2.7	2	2.5	1	3.3
Chest pain	2	1.8	1	1.2	1	3.3
Vomiting	1	0.9	1	1.2	0	0.0
Hemoptysis	1	0.9	1	1.2	0	0.0
Nausea	2	1.8	2	2.5	0	0.0
Unconscious	1	0.9	0	0.0	1	3.3
**Laboratory findings**						
Mean NLR ±SD	6.6 ±7.2		5.3 ±5.4		10.1±10.5	
Mean ALC ±SD	1290 ±664		1349 ±702		1130± 252	
**Type of lesion**						
GGO	75	67.6	53	65.4	22	73.3
Reticular alteration	10	9.0	6	7.4	4	13.3
Fibrotic	1	0.9	0	0.0	1	3.3
Pleural effusion	6	5.4	4	4.9	2	6.7
**Mean RALE score** **±SD**	3.4 ±2.8		2.7 ± 2.7		5.3 ± 2.5	

### Risk factors of mortality

From Table 2, no significant relationship was observed between the outcome of the patients based on gender, type of symptoms, onset of symptoms, ALC score, x-ray abnormalities, and type of lesion. This suggested that those variables have no predictive value for mortality outcome for COVID-19 patients. There is a significant relationship between age, NLR score, lung involvement and RALE score towards the outcome of the patients ( *p*-value <0.05). All of these variables were analyzed using logistic regression, which revealed there are three variables with *p*<0.05: age, NLR score and RALE score ( Table 3).

**Table 2.** Risk factors of COVID-19 hospital mortality.

Variables	Discharged (n=81)		Died (n=30)		*p-*Value
	n	%	n	%	
**Age**					0.009
**≤**50 years	47	58.0	9	30.0	
>50 years	34	42.0	21	70.0	
**Gender**					0.862
Male	39	48.1	15	50.0	
Female	42	51.9	15	50.0	
**Type of symptoms**					0.548
Respiratory	43	53.1	14	46.7	
Non-respiratory	36	44.4	16	53.3	
**Onset of the symptoms**					0.473
≤ 3 days	37	45.7	16	53.3	
>3 days	44	54.3	14	46.7	
**Laboratory findings**					
*NLR Score*					0.002
≤5.8	60	74.1	12	40.0	
>5.8	21	25.9	18	60.0	
*ALC Score*					0.093
≤1000	24	29.6	14	46.7	
>1000	57	70.4	16	53.3	
**CXR abnormalities**					0.055
Negative chest radiography	22	27.2	3	10.0	
Positive chest radiography	59	72.8	27	90.0	
**Lung involvement**					0.007
Unilateral	24	29.6	4	13.3	
Bilateral	35	43.2	23	76.7	
**Type of lesion**					0.430
GGO	53	65.4	22	73.3	
Non-GGO	28	34.6	8	26.7	
**RALE Score**					0.001
≤2	45	55.6	4	13.3	
>2	36	44.4	26	86.7	

**Table 3.** Logistic regression.

	*p*-Value	OR	95% CI
Age	0.043	2.787	1.032-7.531
NLR Score	0.016	3.246	1.241-8.487
Lung involvement	0.965	1.024	0.356-2.946
RALE Score	0.002	6.826	2.076-22.444

Based on the odds ratio (OR) value of each variable, the mortality risk for patients who were of an older age is 2.787 times higher than patients who were of a younger age. A higher NLR score has a 3.246 times higher mortality risk than patients who have lower NLR score. Mortality risk for patients who have a higher RALE score is 6.826 times higher than patients who have lower RALE score. It should be noted that three patients died while showing no abnormalities on their CXR.

### Prediction of mortality

We form a scoring model based on the OR of each variable, which can be seen in Table 4. If the variable were present, we gave the value of “1”, and if absent we gave the value of “0”. We also performed the Hosmer-Lemeshow test and concluded that the model is fit ( *p=*0.802). Based on our scoring model, each score from 111 patients was calculated. The percentage of mortality was then calculated for each score. Table 5 shows that high mortality (>60%) was seen for a total score of 13, while low mortality (<10%) was seen for a total score ≤3.

**Table 4.** Scoring model to predict the mortality of the patient.

Age >50 years	3
NLR Score >5.8	3
RALE Score >2	7

**Table 5.** Probability of mortality based on the total score.

Total Score	Probability of mortality
0	0%
3	6.7%
6	37.5%
7	26.7%
10	38.7%
13	62.5%

**Figure 1.**
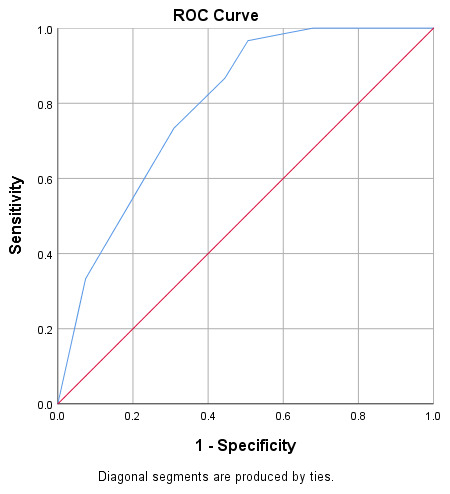
ROC curve analysis for the scoring model to predict the mortality in COVID-19 patients.

The total score was then analyzed with ROC analysis to predict the sensitivity and specificity for the probability of mortality of COVID-19 patients in the hospital. Figure 1 shows that based on the ROC curve, the area under the curve (AUC) of the score is 0.794. The score has a cut off point of 4.5, with a sensitivity of 96.7% and specificity of 49.4%, to predict the mortality of COVID-19 patients in the hospital.

## Discussion

This is the first study to analyze the radiologic and laboratory findings of COVID-19 patients in Indonesia. The main result of our study was that age, NLR score and RALE score have a strong relationship for mortality risk of COVID-19 patients in the hospital.

This study found that the average age of COVID-19 patients who died is 55 ±12.8 years. Another study in Indonesia found a similar result where the average age of COVID-19 patients who died is 58.2 years old ( Rozaliyani *et al*., 2020). This finding is younger than a study in Iraq, who found that the average age of COVID-19 patients who died in hospital is 67.49 ± 15.28 years ( Jalili *et al*., 2020). The younger average age of the patients who died might be due to the co-morbidities that also develop in the relatively younger age of the Indonesian population, especially for type 2 diabetes ( Arifin *et al*., 2019). This study also showed that the mortality risk is three times higher in people age ≥50 years. This finding is similar to a study in the United States, which found that the mortality risk of COVID-19 was 8.1 times higher among those ≥55 years old compared with individuals ages <54 years ( Yanez *et al*., 2020).

Based on our findings, there is no correlation between gender and patient mortality. The result is different from studies in the United States and Iran, which conclude that men has a higher risk of death than women ( Gupta *et al*., 2020; Jalili *et al*., 2020). This could be due to the characteristic of the co-morbidities of the Indonesian population, which affect both men and women at an almost similar number ( Kemenkes, 2019). We also found that there is no correlation between type and onset of symptoms to the outcome of the patients.

There is a correlation between NLR score and the patient outcome. Previously, it was concluded that higher NLR score related to the severe illness of the patients and could be an early identification for patient assessment in the early stage for intensive care unit ( Liu *et al*., 2020; Yang *et al*., 2020). Our study showed that the higher NLR score (>5.8) would increase the risk of mortality by three times. In contrast, we found no correlation between ALC score and patient mortality in the hospital. However, various other research shows that low ALC score is related to disease severity for patients admitted to the hospital for COVID-19 ( Wagner *et al*., 2020) and a higher risk of mortality ( Lippi & Plebani, 2020). 

This study showed that there is no correlation for CXR abnormalities, lung involvement and type of lesion to COVID-19 mortality in the hospital. Interestingly, we also found that three patients (10%) who died showed normal CXR. This could be due to the lower sensitivity of CXR to detect lung lesions compare to CT scan. However, it also shows that COVID-19 could become severe rapidly even though patients have normal CXR on the day of the admission.

Interestingly, we found that RALE score is correlated with patient mortality, and a higher score (>2) will increase mortality risk by seven times. This finding is consistent with another study in the United Kingdom with a larger sample size, which showed that higher radiological severity score (>3) increased the incidence of critical admission or death ( Galloway *et al*., 2020). RALE score in CXR could become a key predictor of mortality because of the simplicity of modality and is broadly available across health care provider.

The prediction score developed in this study demonstrated good accuracy (AUC of 79.4) to predict the discharged probability of COVID-19 patients in the hospital with only three simple parameters. Another score showed good accuracy (AUC of 83) with more complicated parameters, such as health failure, procalcitonin, lactate dehydrogenase, chronic obstructive pulmonary disease, pulse oxygen saturation, heart rate, and age ( Zhao *et al*., 2020). This suggests that our score can be used in a broader setting such as locations with low resource of health care service.

### Limitations

This study only analyzes patient characteristics, and laboratory and radiology findings related to COVID-19 mortality in the hospital. We do not evaluate the co-morbidities of the patient and the hospital setting, such as the location of the treatment where the patient died (emergency room, intensive care, low care), or the resource (facilities, human resource, intervention).

## Conclusions

Age, NLR score and RALE score were associated with mortality of COVID-19 patients in our hospital setting in an Indonesian population. It may be used as a predictor for mortality of COVID-19 patients in the health care centre where radiologists are available. The prediction score may be useful for physicians to determine the mortality risk of a patient with COVID-19.

## Data availability

### Underlying data

Figshare: datasheet f1000research 2909.xlsx, https://doi.org/10.6084/m9.figshare.13017899.v1 ( Sensusiati, 2020).

Data are available under the terms of the Creative Commons Attribution 4.0 International license (CC-BY 4.0).
