# Supplementary material for: Age, neutrophil lymphocyte ratio, and radiographic assessment of the quantity of lung edema (RALE) score to predict in-hospital mortality in COVID-19 patients: a retrospective study
Source: F1000Res. 2021 Jan 6;9:1286. Originally published 2020 Oct 30. [Version 2] doi: 10.12688/f1000research.26723.2 (PMC7836085; doi:10.12688/f1000research.26723.2)
Supplement: Peer review history

# Peer review: Reviewer response for version 2

**DOI:** 10.5256/f1000research.33024.r76665

**Published:** 2021-01-25

**Authors:** Wiwien Heru Wiyono

**Affiliations:** 1 Department of Pulmonology and Respiratory Medicine, Faculty of Medicine, Universitas Indonesia, Jakarta, Indonesia

I have read the revised version and I agree to all revisions so I approved this article.

Is the work clearly and accurately presented and does it cite the current literature?

Yes

If applicable, is the statistical analysis and its interpretation appropriate?

Yes

Are all the source data underlying the results available to ensure full reproducibility?

Partly

Is the study design appropriate and is the work technically sound?

Yes

Are the conclusions drawn adequately supported by the results?

Yes

Are sufficient details of methods and analysis provided to allow replication by others?

Yes

Reviewer Expertise:

Pulmonary medicine

I confirm that I have read this submission and believe that I have an appropriate level of expertise to confirm that it is of an acceptable scientific standard.

# Peer review: Reviewer response for version 1

**DOI:** 10.5256/f1000research.29506.r74043

**Published:** 2020-11-20

**Authors:** Wiwien Heru Wiyono

**Affiliations:** 1 Department of Pulmonology and Respiratory Medicine, Faculty of Medicine, Universitas Indonesia, Jakarta, Indonesia

There are some statements in this paper that are not so accurate.

**Introduction** **:**

 My comment:

 To reduce the mortality rate in some hospitals in Surabaya is not by finding characteristics and risk factors to predict mortality. As I know at that time tracing in Surabaya still low in subjects, so the CFR in this city is still higher than Jakarta and the mean CFR in Indonesia.

 "the purpose of this study was to assess the factors associated with COVID-19 mortality in hospital patients and develop a prediction score based on these findings."

 My comment:

 To assess the factors associated with mortality even to develop prediction score should use all factors. But this study actually only uses some parameters not include commorbidities.

**Discussion :**

 The statement:

1. " …….this finding is similar to a study in the US, which found that an older age (> 60 years) is associated with a higher risk of death  is not so accurate, because:"

My comments:

1. The difference between mean ages of >50 and >60 is so significant.
2. Criteria for elder people in Indonesia is >60 years old.
3. The younger average of the patients in this study is not representative of Indonesia Covid patients.

 The statement :

1. "CXR could become a key predictor of mortality because………."

  My comment:

1. This statement is over confident and is in contradiction with the statement: "This study showed that there is no correlation for CXR abnormalities……"

**Conclusion**:

 The statement:

1. "It may be used as a predictor for mortality of COVID 19 patients in low health care resource setting."

My comment :

1. It is not so true. The RALE  score should be made by radiologists who have many experiences, so it only applicable in a referral hospital setting.

Is the work clearly and accurately presented and does it cite the current literature?

Yes

If applicable, is the statistical analysis and its interpretation appropriate?

Yes

Are all the source data underlying the results available to ensure full reproducibility?

Partly

Is the study design appropriate and is the work technically sound?

Yes

Are the conclusions drawn adequately supported by the results?

Yes

Are sufficient details of methods and analysis provided to allow replication by others?

Yes

Reviewer Expertise:

Pulmonary medicine

I confirm that I have read this submission and believe that I have an appropriate level of expertise to confirm that it is of an acceptable scientific standard, however I have significant reservations, as outlined above.

# Response

**Published:** 2020-12-21

**Authors:** Anggraini Dwi Sensusiati

**Affiliations:** Airlangga University, Indonesia

The authors thank the Honorable Reviewer 2 for the excellent review of the paper and in-depth suggestions made to improve the quality of the paper. Followings are the responses to the suggestions of the Honorable Reviewer. The valuable suggestions and corrections are incorporated cautiously, and the paper is made clearer while revising the manuscript.

**Introduction:**

 Comment:

 To reduce the mortality rate in some hospitals in Surabaya is not by finding characteristics and risk factors to predict mortality. As I know at that time tracing in Surabaya still low in subjects, so the CFR in this city is still higher than Jakarta and the mean CFR in Indonesia.

 *We acknowledged the possible cause of high fatality rate and added the sentence "Several factors may associate with the higher CFR such as low testing, low contact tracing and poor COVID-19 register (Sorci *et al., *2020). However, finding characteristics and risk factors to predict mortality is of utmost importance in order to prevent the mortality."

**"the purpose of this study was to assess the factors associated with COVID-19 mortality in hospital patients and develop a prediction score based on these findings."**

 Comment:

 To assess the factors associated with mortality even to develop prediction score should use all factors. But this study actually only uses some parameters not include commorbidities.

 *We acknowledged the study limitation which did not evaluate the commorbidities in the limitations section. We focused the research on the demographical characteristic, laboratory and radiological findings associated with COVID-19 mortality in hospital patients and develop a prediction score based on these findings.

**Discussion :**

 The statement:

**" …….this finding is similar to a study in the US, which found that an older age (> 60 years) is associated with a higher risk of death  is not so accurate, because:"**

**C**omments:

1. The difference between mean ages of >50 and >60 is so significant.
2. Criteria for elder people in Indonesia is >60 years old.
3. The younger average of the patients in this study is not representative of Indonesia Covid patients.

*We found similar finding in another research in Indonesia where the average age of COVID-19 patients who died is 58.2 years old (Rozaliyani *et al., *2020). We also found that this finding is similar to a study in the United States, which found that the mortality risk of COVID-19 was 8.1 times higher among those ≥55 years compared with individuals ages <54 years (Yanez *et al.,* 2020).

 The statement :

 " **CXR could become a key predictor of mortality because………."** 

 Comment:

1. This statement is over confident and is in contradiction with the statement: "This study showed that there is no correlation for CXR abnormalities……"

*We meant that the RALE score from CXR might become a key predictor 

**Conclusion:**

 The statement:

**"It may be used as a predictor for mortality of COVID 19 patients in low health care resource setting."**

 Comment:

1. It is not so true. The RALE  score should be made by radiologists who have many experiences, so it only applicable in a referral hospital setting.

*We changed the "low health care resource setting" into "health care centre where radiologists are available."

 We thank Prof. Wiwien for raising these points which we feel have improved the paper considerably.

*Sensusiati AD et al.*

# Peer review: Reviewer response for version 1

**DOI:** 10.5256/f1000research.29506.r74045

**Published:** 2020-11-20

**Authors:** Hamzaini Abdul Hamid

**Affiliations:** 1 Department of Radiology, Faculty of Medicine, Universiti Kebangsaan Malaysia Medical Centre (UKMMC), Kuala Lumpur, Malaysia

Congratulation and I think the paper has been presented very well. The statistical analysis and tools were done appropriately. 

 This paper definitely will contribute to the management of COVID 19 patients i.e. in predicting which patient may deteriorate and need to be monitor closely.

 Although in this paper there is no correlation for CXR abnormalities, lung involvement and type of lesion to COVID-19 mortality. However, I think all the above CXR changes would also contribute collectively to the RALE score. 

 Overall, well done and congratulation to the author. Just a minor error in spelling.

Is the work clearly and accurately presented and does it cite the current literature?

Yes

If applicable, is the statistical analysis and its interpretation appropriate?

Yes

Are all the source data underlying the results available to ensure full reproducibility?

Yes

Is the study design appropriate and is the work technically sound?

Yes

Are the conclusions drawn adequately supported by the results?

Yes

Are sufficient details of methods and analysis provided to allow replication by others?

Yes

Reviewer Expertise:

General Radiology, Paediatric Radiology

I confirm that I have read this submission and believe that I have an appropriate level of expertise to confirm that it is of an acceptable scientific standard.
